# Four coding-complete genomes of bovine viral diarrhea virus-2a (*Orthopestivirus tauri* genotype a) isolated from farmed white-tailed deer (*Odocoileus virginianus*) in Florida, USA, in 2018

**DOI:** 10.1128/mra.00508-26

**Published:** 2026-09-16

**Authors:** An-Chi Cheng, Pedro H. de Oliveira Viadanna, Zoe S. White, Tracey L. Moquin, Julia C. Loeb, Olivia F. Goodfriend, Kristen N. Wilson, John A. Lednicky, João H. Jabur Bittar, Thomas B. Waltzek, Samantha M. Wisely, Kuttichantran Subramaniam, Juan M. Campos Krauer

**Affiliations:** 1 Department of Large Animal Clinical Sciences, College of Veterinary Medicine, University of Florida 3463, Gainesville, Florida, USA; 2 Department of Infectious Diseases and Immunology, College of Veterinary Medicine, University of Florida 3463, Gainesville, Florida, USA; 3 Emerging Pathogens Institute, University of Florida 145775, Gainesville, Florida, USA; 4 Department of Fish and Wildlife Sciences, College of Natural Resources, University of Idaho 680952, Moscow, Idaho, USA; 5 Department of Wildlife Ecology and Conservation, Institute of Food and Agricultural Sciences, University of Florida 440202, Gainesville, Florida, USA; 6 Department of Environmental and Global Health, College of Public Health and Health Professions, University of Florida 271714, Gainesville, Florida, USA; 7 Washington Animal Disease Diagnostic Laboratory 70122, Pullman, Washington, USA; 8 Department of Veterinary Microbiology and Pathology, College of Veterinary Medicine, Washington State University 6760, Pullman, Washington, USA; Katholieke Universiteit Leuven, Leuven, Belgium

**Keywords:** white-tailed deer, *Odocoileus virginianus*, bovine viral diarrhea virus, BVDV, *Orthopestivirus tauri*, *Orthopestivirus*, *pestivirus*, deer farming, disease surveillance

## Abstract

To date, no bovine viral diarrhea virus-2 (BVDV-2) coding-complete genomes are available from white-tailed deer (WTD) in Florida. We determined the coding-complete genome sequences of four BVDV-2 isolates from farmed WTD that were found dead in 2018. These belonged to BVDV-2 sub-genotype a.

## ANNOUNCEMENT

Bovine viral diarrhea virus (BVDV, genus *Orthopestivirus*, family *Pestiviridae*) has a single-stranded, positive-sense RNA genome (12.3 kb) and infects mammals, including cattle, deer, and swine. BVDV-1 (*Orthopestivirus bovis*, formerly *Pestivirus bovis* and *Pestivirus A*) has been classified into at least 21 sub-genotypes and BVDV-2 (*Orthopestivirus tauri*, formerly *Pestivirus tauri* and *Pestivirus B*) into four sub-genotypes: BVDV-2a to BVDV-2d (1). In white-tailed deer (WTD; *Odocoileus virginianus*), infections by BVDV can lead to abortion, delivery of mummified fetuses, depression, lethargy, and pyrexia (2–8).

Three farmed WTD does (OV853, OV854, and OV858) and one buck (OV856) found dead in Florida in 2018 (Table 1) were field-necropsied, and tissues were collected as previously described (9). Virus isolation attempts from spleen homogenates in BHK-21, C6/36, and Vero E6 cells (10) were unsuccessful. Virus-induced cytopathic effects (CPEs) were observed in OHH1.K cells and *Bos taurus* (BT) cells. After CPE was evident in more than 80% of cells (18 dpi), RNA was extracted from virions in the spent cell culture media of BT cells using a QIAamp Viral RNA Mini Kit (Qiagen, USA), next-generation sequencing libraries constructed using an NEBNext Ultra RNA Library Prep Kit (New England Biolabs, USA), and genomic sequencing performed using a 600-cycle v3 kit on an Illumina MiSeq sequencer (Illumina, USA). Paired-end FASTQ files were generated using the Illumina GenerateFASTQ workflow in MiSeq Control Software v2.6.2.1. No additional quality control tools were used prior to downstream analyses.

**TABLE 1:** Demographics of farmed Florida white-tailed deer (WTD) found dead with BVDV-2a infection and corresponding viral sequence information

Animal ID	Age (year)	Sex	Farm ID	County	Clinical signs	Necropsy date (mo-day-yr)	Total sequencing reads	Average genome coverage (reads/nucleotide)
OV853	1.5	Female	Farm A	Martin	No clinical signs prior to being found dead	9-10-2018	4,408,898	150
OV854	1.5	Female	Farm A	Martin	No clinical signs prior to being found dead	9-10-2018	NA^*a*^	NA
OV856	4	Male	Farm B	Lafayette	No clinical signs prior to being found dead	9-14-2018	4,304,312	2,676
OV858	2	Female	Farm A	Martin	No clinical signs prior to being found dead	9-12-2018	NA	NA

^*a*^ NA, not applicable.

**Fig 1:**
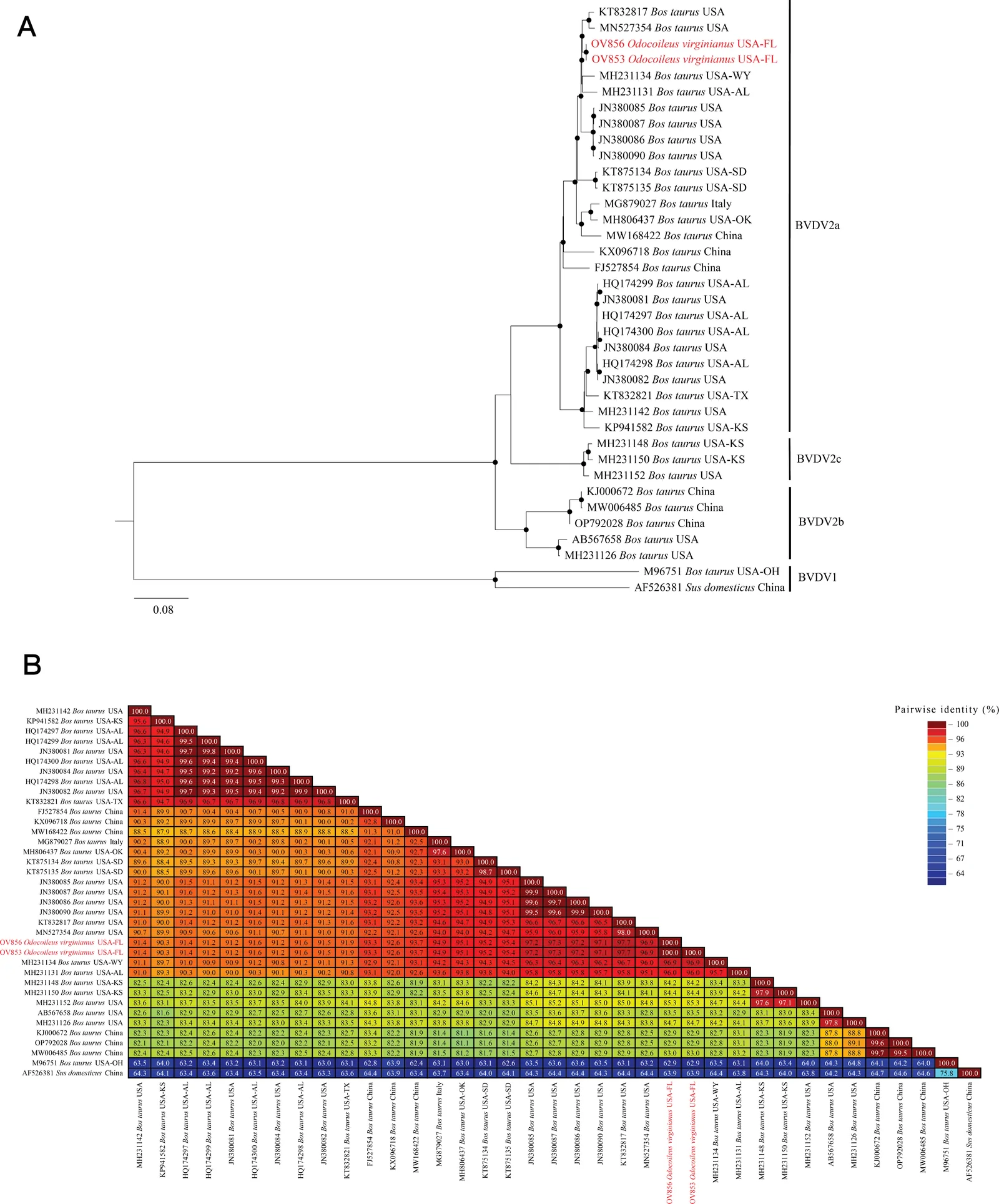
Phylogenetic tree and sequence identity matrix of BVDV-2a isolated from Florida farmed WTD compared to 2 BVDV-1 and 33 BVDV-2 reference strains. (**A**) The phylogenetic tree illustrates the relationships of BVDV-2a isolates from farmed WTD in Florida (OV853 and OV856) to BVDV-1 and BVDV-2 reference sequences. The genome sequences were aligned using CLC Genomics Workbench v2.0 (Qiagen, USA). The phylogenetic tree was generated based on the nucleotide sequence alignment of the polyprotein and constructed using the maximum likelihood method in IQ-TREE (14) with 1,000 non-parametric standard bootstraps performed to test the robustness of the clades. Nodes marked with black circles indicate bootstrap values ≥ 80%. For each reference sequence, the GenBank accession number, host species, and the geographic region where the strains were identified are provided. (**B**) Sequence identity matrix of BVDV *E2* glycoprotein gene. Pairwise genetic comparisons of the nucleotide sequences of the *E2* glycoprotein gene were performed using Sequence Demarcation Tool v1.2 (15) by aligning the *E2* glycoprotein gene sequences (1,117 bp) from OV853 and OV856 BVDV isolates with the BVDV-1 and BVDV-2 reference sequences.

Host sequences (from BT cells [*Bos taurus*; GenBank assembly number: GCA_905123515]) were removed from the sequencing data using Kraken 2 (11). Paired-end DNA reads were *de novo* assembled using SPAdes v3.15.3 (12). The assembled contigs were identified as BVDV-2 by BLASTn searches against the National Center for Biotechnology Information (NCBI) nucleotide database and subsequently classified as BVDV-2a by phylogenetic analysis. Genome annotation was conducted using GeneMarkS v4.28 (13). A total of 35 BVDV-1 and BVDV-2 complete genome sequences or complete coding sequences were retrieved from the NCBI GenBank database for use as reference sequences. The genome sequences were aligned using CLC Genomics Workbench v2.0 (Qiagen, USA). A phylogenetic tree was generated using IQ-TREE (14) (Fig. 1A). Pairwise genetic comparisons of the nucleotide sequences of the *E2* glycoprotein gene were performed using Sequence Demarcation Tool v1.2 (15) (Fig. 1B). Default parameters were used except where otherwise noted. Next-generation sequencing yielded a coding complete 12,273 rnt long BVDV-2a genome from the four animals, with GC contents of 44.96%. The genomic sequences of OV853, OV854, and OV858 were identical, while OV856 exhibited a single mismatch at nucleotide position 1,674, wherein adenine (A) was substituted with guanine (G), resulting in a nonsynonymous substitution of the amino acid from valine to methionine in the polyprotein.

Genomic analyses revealed that the BVDV-2a sequences from our WTD clustered with BVDV-2a sequences from cattle in the United States. This raises the possibility of cross-species transmission of BVDV-2a between the farmed WTD and cattle at adjacent ranches, as it is known that BVDV can be transmitted through direct contact from cattle to WTD and vice versa (16, 17).

## Data Availability

The coding-complete gene coding sequences for all genomes recovered in this study have been deposited in the NCBI GenBank database and are available under the GenBank accession number PV541660.1 and PV541661.1. Raw reads are available under SRR38484040 and SRR38484039.
